# New Natural Diterpene-Type Abietane from *Tetradenia riparia* Essential Oil with Cytotoxic and Antioxidant Activities

**DOI:** 10.3390/molecules19010514

**Published:** 2014-01-03

**Authors:** Zilda Cristiani Gazim, Felipe Rodrigues, Ana Carolina Lourenço Amorin, Cláudia Moraes de Rezende, Marina Soković, Vele Tešević, Ivan Vučković, Gordana Krstić, Lucia Elaine Ranieri Cortez, Nelson Barros Colauto, Giani Andrea Linde, Diógenes Aparício Garcia Cortez

**Affiliations:** 1 Postgraduate Program in Biotechnology Applied to Agriculture, University, Umuarama, PR 87.502-210, Brazil; E-Mails: cristianigazim@unipar.br (Z.C.G.); nbc@unipar.br (N.B.C.); gianilinde@unipar.br (G.A.L.); 2 Laboratory of Experimental Oncology, Federal University of Ceará, Fortaleza, CE 60.455-970, Brazil; E-Mail: feliperbio@yahoo.com.br; 3 Technology Center, Institute of Chemistry, Federal University of Rio de Janeiro, Rio de Janeiro, RJ 21941-909, Brazil; E-Mails: anamorim13@hotmail.com (A.C.L.A.); claudia.rezendeufrj@gmail.com (C.M.R.); 4 Institute for Biological Research, University of Belgrade, Belgrade 11000, Serbia; E-Mail: mris@ibiss.bg.ac.rs; 5 Faculty of Chemistry, University of Belgrade, Belgrade 11000, Serbia; E-Mails: vtesevic@chem.bg.ac.rs (V.T.); ivuckovic@chem.bg.ac.rs (I.V.); gordana1988@gmail.com (G.K.); 6 Postgraduate Program in Health Promotion, University Center of Maringá, Maringá, PR 87050-390, Brazil; E-Mail: luciaelaine@cesumar.br; 7 Postgraduate Program in Pharmaceutical Sciences, State University of Maringá, Maringá, PR 87020-900, Brazil

**Keywords:** *Tetradenia riparia*, essential oil, abietane diterpenes, 9β,13β-epoxy-7-abietene, 6,7-dehydroroyleanone, cytotoxicity activity, antioxidant activity

## Abstract

*Tetradenia riparia* (Hochstetter) Codd belongs to the Lamiaceae family and it was introduced in Brazil as an exotic ornamental plant. A previous study showed its antimicrobial, acaricidal and analgesic activities. Two compounds were isolated from essential oil of *T. riparia* leaves and identified as 9β,13β-epoxy-7-abietene (**1**), a new one, and 6,7-dehydroroyleanone (**2**), already reported for another plant. The structure of these compounds was determined by spectroscopic analysis and by comparison with literature data. The cytotoxic activities of the essential oil and compounds **1** and **2** were determined by a 3-(4,5-dimethylthiazol-2-yl)-2,5-diphenyl-2H-tetrazolium bromide (MTT) assay, and by tumor cells MDA-MB-435 (human breast carcinoma), HCT-8 (human colon), SF-295 (human nervous system) and HL-60 (human promyelocytic leukemia). The essential oil and compound **1** showed high cytotoxic potential of the cell lines SF-295 (78.06% and 94.80%, respectively), HCT-8 (85.00% and 86.54%, respectively) and MDA-MB-435 (59.48% and 45.43%, respectively). Compound **2** had no cytotoxic activity. The antioxidant activity was determined by 2,2-diphenyl-1-picryl-hydrazyl (DPPH), β-carotene-linoleic acid system and 2,2'-azinobis-(3-ethylbenzothiazoline-6-sulfonic acid) (ABTS) assays. The inhibitory concentration (IC_50_ in µg mL^−1^) for essential oil and compound **2** was, respectively 15.63 and 0.01 for DPPH; 130.1 and 109.6 for β-carotene-linoleic acid and 1524 and 1024 for ABTS. Compound **1** had no antioxidant activity. By fractioning the oil, it was possible to identify two unpublished compounds: **1** with high cytotoxic potential and **2** with high antioxidant potential.

## 1. Introduction

*Tetradenia riparia* (Hochstetter) Codd, also known as *Iboza riparia* N. E. BR., *Moschosma riparium* or *Tetradenia riparia* (Hochstetter) N. E. BR., belongs to the Lamiaceae family and is native to South Africa, where it is one of the most aromatic and popular medicinal plants [1,2,3,4,5]. This exotic plant is popularly known as false myrrh, lemon verbena, lavandula, misty plume, or incense. In Brazil, *T. riparia* was introduced as an exotic ornamental plant and is cultivated in parks, gardens, homes, and botanical gardens where it releases a very intense and pleasant aroma [6].

The Lamiaceae family has been studied to improve the production of essential oils and identify the compounds of these oils [7]. Gazim *et al.* [8] report that the essential oil of *T. riparia* is a complex mixture of terpenoids: monoterpenes, sesquiterpenes, and diterpenes (hydrocarbons or oxygenated) and the most representative class of the oil composition is the oxygenated sesquiterpenes, especially 14-hydroxy-9-*epi*-caryophyllene.

From the essential oil of *T. riparia*, Zelnik *et al.* [6] isolated ibozol and 7α-hydroxyroyleanone, Van Puyvelde *et al.* [4,9] isolated diterpenediol 8(14),15-sandaracopimaradiene-7α,18-diol, and 1',2'-dideacetylboronolide. The compounds 8(14),15-sandaracopimaradiene-2α,18-diol, a diterpenod with antimicrobial activity [5], 5,6-dehydro-α-pyrone (umuravumbolide) [10] and tetradenolide α-pyrone [5] have also been isolated. Although there is broad report on the isolated compounds of *T. riparia* essential oil, our group still searches for minor constituents that represent a great challenge for researchers. The essential oil of *T. riparia* present biological activities that are reported in the literature as antispasmodic [4], larvicidal and insecticidal [1], anti-mycobacterial [11], antimalarial [2], repellent of *Anopheles gambiae* [12], antimicrobial, antinociceptive [8], and acaricidal against *Rhipicephalus* (*Boophilus*) *microplus* [13].

In recent years, the extracts and the essential oils of many plants have been screened for their antioxidant activities. Evaluation of antioxidant activities as natural food additives is very important because some plants have abilities to scavenge free radicals produced in the human body [14]. The fact that, *in silico*, some compounds behave like antioxidants does not at all predict their biological effects in living cells [15]. Fan and Lou [16] described that some polyphenols were good antioxidants at low concentration but, at higher concentration, they induced cellular DNA damage. Nevertheless, retinol and tocopherol have antioxidant and antimutagenic activities at low concentration but, at high concentration, they become genotoxic [17]. Thus, the cytotoxic activity has been evaluated with the antioxidant activity of essential oils to better understand its biological activity. Cytotoxicity analysis using the 3-(4,5-dimethylthiazol-2-yl)-2,5-diphenyl-2H-tetrazolium bromide (MTT assay has been utilized in the screening program of the United Stated National Cancer Institute (NCI), which tests more than 10,000 samples a year [18]. This method can analyze the viability and the metabolic condition of the cell and can determine essential oil cytotoxicity [19]. In this paper, we describe the isolation and identification of a new compound from *T. riparia* essential oil, andcorresponding *in vitro* antioxidant and cytotoxic activities. 

## 2. Results and Discussion

### 2.1. Identification of the New 9β,13β-Epoxy-7-abietene (**1**) and an 6,7-Dehydroroyleanone (**2**) Compounds

Compound **1**, white amorphous powder, melting point (61–63 °C), showed a molecular ion peak at *m/z* 288 [M^+^] in its electron ionization (EI) mass spectrum and HR-MS (ESI) [M+H]^+^ Found *m/z*: 289.2525 (calc. for C_20_H_33_O: 289.2532), which matches the proton (^1^H) and carbon-13 (^13^C) nuclear magnetic resonance (NMR) data, including distortionless enhancement by polarization transfer (DEPT) spectra, suggested a molecular formula C_20_H_32_O. This molecular formula indicated five degrees of unsaturation.

The ^1^H and ^13^C-NMR spectra showed 20 carbon signals, including three quaternary and two tertiary methyls [*δ*H 1.00 (3H, s, Me-20), 0.87 (3H, s, Me-18), 0.95 (3H, s, Me-19), 0.96 (3H, d, *J* = 7.0 Hz, Me-16), 0.93 (3H, d, *J* = 7.0 Hz, Me-17); *δ*C 15.5 (C-20), 33.5 (C-18), 22.3 (C-19), 18.2 (C-17) 18.0 (C-16)], two methines [*δ*H 2.09 (1H, sep., *J* = 7.0 Hz, H-15); *δC* 32. 8 and *δ*H 1.38 (1H, dd., *J* = 5.0; 10 Hz, H-5); *δ*C 45. 7] and one olefinic bond [*δH* 5.25 (1H, m, H-7); *δ*C 113.8 / *δ*C 142.4 (C-8)], seven methylenes, and four quaternary carbons signals (Table 1).

**Figure 1:**
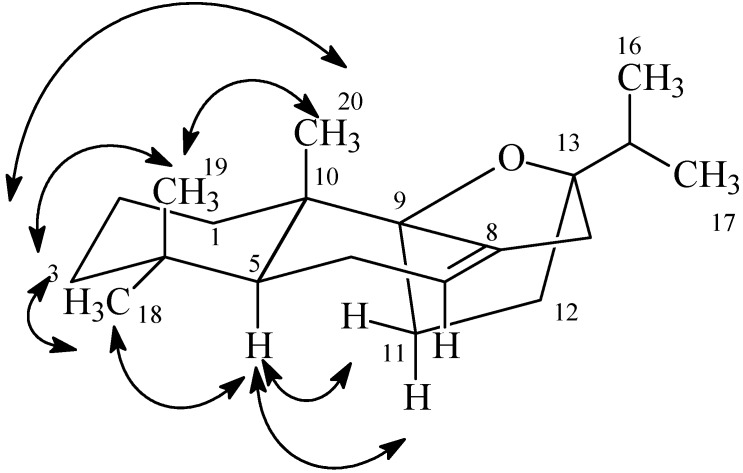
COSY(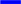) and selected HMBC (H→C) correlations of **1**.

The ^1^H-NMR and ^1^H-^1^H COSY spectra revealed the presence of four separate *J*-coupling networks (**A**–**D**): **A**: –CH_2_–CH_2_–CH_2_– (*δ*_H_ 1.52 m, 2.02 m and 1.46 m, 1.61 m and 1.18 m, 1.42 m); **B**: –CH–CH_2_–CH = (*δ*_H_ 1.38 dd and 1.92, 2.05 and 5.25 m); **C**: –CH_2_–CH_2_–(*δ*_H_ 1.96 m, 1.56 m and 1.58 m); **D**:–CH(CH_3_)_2_ (*δ*_H_ 0.96 d, 0.93 d and 2.09 sep.). This information, along with the heteronuclear multiple-bond correlation spectroscopy HMBC correlations traced from five methyls (Me-16,17,18,19,20), suggested the presence of an abietane skeleton (Figure 1).

**Table 1:** ^1^H- and ^13^C-NMR data of compound **1** [500 MHz (^1^H), 125 MHz (^13^C), tetramethylsilane (TMS) and deuterated chloroform (CDCl3)].

Position	^1^H [δ(ppm), mult, *J* in Hz]	^13^C [δ(ppm)]
1α	2.02 m	33.3
1β	1.52 m	33.3
2α	1.61 m	18.5
2β	1.46 m	18.5
3α	1.42 m	42.7
3β	1.18 m	42.7
4	-	33.3
5	1.38 dd (5.0; 10)	45.7
6α	2.05 m	24.6
6β	1.92 m	24.6
7	5.25 m	113.8
8	-	142.4
9	-	90.8
10	-	36.6
11α	1.96 m	30.1
11β	1.56 m	30.1
12	1.58 m	33.1
13	-	87.9
14	2.11 m	38.0
15	2.09 sep. (7)	32.8
16	0.96 d (7)	18.0
17	0.93 d (7)	18.2
18	0.87 s	33.5
19	0.95 s	22.3
20	1.00 s	15.5

**Figure 2:**
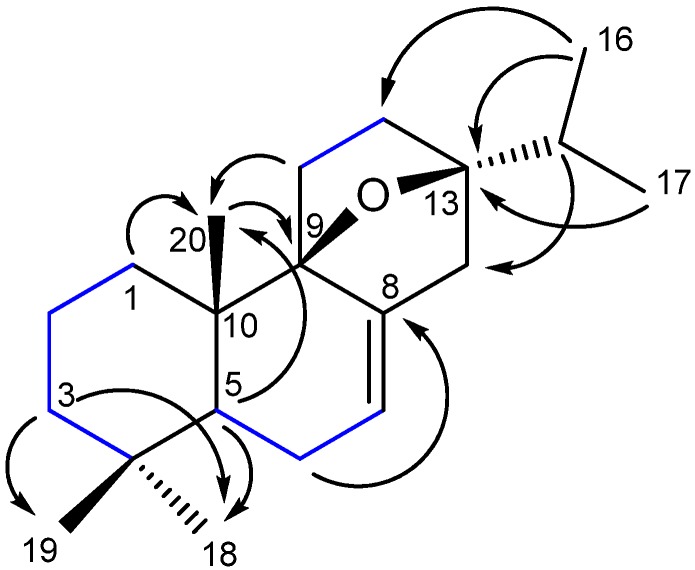
Selected NOESY correlations of **1**.

**Figure 3:**
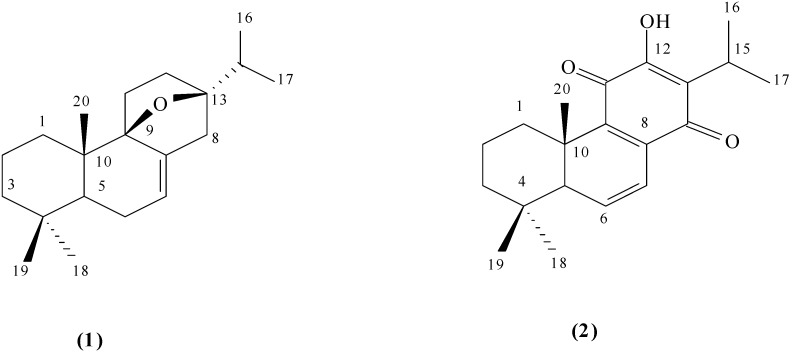
Structural formula of compounds 9β,13β-epoxy-7-abietene (**1**) and 6,7-dehydroroyleanone (**2**).

The presence of two oxygen-bearing quaternary carbons (*δ*C 87.9 and *δ*C 90.8) in ^13^C-NMR spectrum and the multiplicity in the ^1^H-NMR of the isopropyl methine signals, showing couplings with methyl only, indicated an epoxide bridge between the C-9 and C-13 positions. This assumption was further supported by the HMBC spectrum. In the HMBC spectrum (Figure 1), the proton signal at *δ*H 1.00 (H-20) was correlated with the signal at *δ*C 90.8 (C-9), and the proton signals at *δ*H 0.96 and 0.93 (H-16 and H-17) correlated with the signal at *δ*C 87.9 (C-13), respectively. Based on NOE correlations of H_3_-19/H_3_-20 and H_3_-18/H-5/H-11 (Figure 2) the complete structure of the compound **1** was elucidated as 9β,13β-epoxy-7-abietene (Figure 3). Compound **2** gave an [M − H]^−^ at *m/z* 313 and it was identified by ESI-MS and NMR as 6,7-dehydroroyleanone (Figure 3). Spectral data corresponded with data published previously [20].

**Table 2:** Growth inhibition percentageof *T. riparia* essential oil and isolated compounds 9β,13β-epoxy-7-abietene and 6,7-dehydroroyleanone of three tumoral cell lines at a single dose of 50 µg mL^−1^ for the essential oil and 25 µg mL^−1^ for the isolated compounds.

Isolated compounds	Growth inhibition (%) of cell line *		
	MDA-MB-435	SF-295	HCT-8
*T. riparia* essential oil	59.48 ± 0.51 ^a^	78.06 ± 0.67 ^b^	85.00 ± 0.46 ^a^
9β,13β-epoxy-7-abietene	45.43 ± 1.36 ^b^	94.80 ± 0.82 ^a^	86.54 ± 1.37 ^a^
6,7-dehydroroyleanone	3.34 ± 0.11 ^c^	15.30 ± 0.07 ^c^	12.08 ± 0.31 ^b^

* MDA-MB-435 = human melanoma cell line, SF-295 = nervous system human cell line and HCT-8 = human colon cell line. Values (%) are averages ± standard deviation of averages. The averages followed by the same letter in the same column do not differ statistically by Tukey’s test (*p* < 0.01).

### 2.2. Cytotoxic Analysis

The cytotoxicity assays for the essential oil and isolated compounds 9β,13β-epoxy-7-abietene and 6,7-dehydroroyleanone are presented in Table 2, with their respective percentage of inhibition.

The results showed a 78.06% and 94.80% potential inhibition of the essential oil and 9β,13β-epoxy-7-abietene for tumoral cell lines SF-295, respectively, and 85.00% and 86.54% for HCT-8, respectively. It indicates a high cytotoxic potential of this essential oil and fraction for these two tumor cell lines, since the inhibition values were above 75%. For the MDA-MB-435 (human melanoma cell) strain, the cytotoxic potential was 59.48% and 45.43%, respectively, considering the low inhibitory activity of the cellular growth. The compound 6,7-dehydroroyleanone did not have cellular activity for the tested lines. The samples were evaluated within a determined scale following the adopted classifications for any type of cytotoxic assay according to the current international standards: without activity (1%–20% inhibition of observed cellular growth), with little activity (inhibitions of cellular growth, varying from 20%–50%), with moderate activity (inhibition of the cellular growth varying from 50%–70%), with high activity (growth inhibition varying from 70% to 100%) [21].

### 2.3. Antioxidant Analysis

The antioxidant activities of *T. riparia* essential oil, 9β,13β-epoxy-7-abietene and 6,7-dehydroroyleanone were evaluated using DPPH radical scavenging, β-carotene-linoleic acid and 2,2'-azinobis-(3-ethylbenzothiazoline-6-sulfonic acid) (ABTS) assays. The 9β,13β-epoxy-7-abietene showed no antioxidant activity in all antioxidant assays. The 6,7-dehydroroyleanone had higher (*p* ≤ 0.01) antioxidant activity than essential oil, quercetin or butylated hydroxytoluene (BHT) controls for DPPH and β-carotene-linoleic acid assays but not to ABTS assay (Table 3). For DPPH method the IC_50_ (µg mL^−1^) of 6,7-dehydroroyleanone was around 1500 and 200 times lower than essential oil and quercetin, respectively (Table 3), that indicates higher (*p* ≤ 0.01) antioxidant activity for the isolated compound.

**Table 3:** Values for antioxidant concentration that reduces 50% of the free radical concentration (Inhibitory Concentration; IC50; µg mL^−1^) for *T. riparia* essential oil and isolated compound 6,7-dehydroroyleanone using three different methods: DPPH radical scavenging, β-carotene-linoleic acid or ABTS [2,2'-azinobis-(3-ethylbenzothiazoline-6-sulfonic acid)] assay.

Compounds	IC_50_ (µg mL^−1^)		
	DPPH	β-Carotene-linoleic acid	ABTS
*T. riparia* essential oil	15.63 ± 0.25 ^a^	130.1 ± 5.76 ^a^	1524 ± 123 ^a^
6,7-Dehydroroyleanone	0.010 ± ˂0.001 ^b^	109.6 ± 3.83 ^b^	1024 ± 54 ^b^
BHT	-	133.5 ± 7.36 ^a^	-
Quercetin	2.05 ± 0.02 ^c^	-	190 ± 38 ^c^

Values are averages (n = 4) ± standard deviation. The averages followed by the same letter in the same column do not differ statistically by Tukey’s test (*p* < 0.01).

Until the present date, no studies have been carried out to determine the antioxidant activity of the essential oil and the isolated fractions of *T. riparia* oil. As described by Suhaj [22] the oxidation is one of the major causes of chemical spoilage, and it promoted an increasing interest in the industry and scientific research for compounds with strong antioxidant properties. Thus, researchers have tried to isolate compounds with high antioxidant activity. There is a long list of antioxidant compounds such as ascorbic acid, β-carotene, ubiquinone, tannins, *etc*. [23], used as positive controls, but quercetin is one of the most common. Modern consumers ask for natural products, free of synthetic additives. According to the results of this study, it is clearly indicated that 6,7-dehydroroyleanone has higher antioxidant activity than quercetin. This compound could be used as a source of natural antioxidants and as a possible food supplement or in pharmaceutical industry.

## 3. Experimental

### 3.1. General

The NMR spectra (^1^H, ^13^C, DEPT, HSQC, HMBC and NOESY) were recorded on a Bruker Avance III 500 spectrometer (500.26 for ^1^H and 125.80 MHz for ^13^C), with CDCl_3_ as solvent and tetramethylsilane (TMS) as reference. Gas-chromatographic (GC) and mass-spectrometric (MS) analysis was performed using a Agilent 5973 Network chromatograph coupled to a Agilent 5973 MSD spectrometer (Agilent Technologies, Santa Clara, CA, USA), High-resolution ESI-MS were recorded on a Thermo Scientific LTQ Orbitrap XL mass spectrometer. All MS spectra experiments were acquired in ESI positive ion mode. Full scan spectra were performed over a scan range of *m/z* 100–1000 (Thermo Fisher Scientific, Waltham, MA, USA), silica gel 60 (70–230 and 230–400 mesh) and thin layer chromatography (TLC): silica gel plates F_254_ (0.25 mm in thickness).

### 3.2. GC Analysis

The separation was achieved using an Agilent 19091S-433 HP-5MS fused silica capillary column, 30 m × 0.25 mm i.d. (internal diameter of the column), 0.25 µm film thickness. GC oven temperature was programmed from 60 °C to 285 °C at a rate of 4.3 °C min^−1^. Helium was used as carrier gas; inlet pressure was 25 kPa; linear velocity: 1 mL min^−1^ at 210 °C. Injector temperature: 250 °C. Injection mode: splitless. MS scan conditions: source temperature, 200 °C; interface temperature, 250 °C; E energy, 70 eV; mass scan range, 40–350 amu.

### 3.3. Plant Material

*T. riparia* leaves were collected monthly from September 2006 to August 2007 in Umuarama, state of Paraná, Brazil (−23°45'59 S, −53°19'30 W, 391 m). The specimen was identified by Ezilda Jacomasi, Ph.D. in botany, responsible for the Herbarium of the Paranaense University, Umuarama, Paraná, Brazil, and the specimen voucher was deposited on the number 2502. The leaves were collected at 6:30 a.m. and 8:00 a.m., respectively. The hydrodistillated oil was obtained using a Clevenger apparatus and filtered with anhydrous Na_2_SO_4_, and stored in a freezer during the experiment period. The distillations were performed in triplicate.

### 3.4. Essential Oil Fractionation of the Leaves

The essential oil (2 g) from *T. riparia* leaves was submitted to column chromatography over a silica gel support and eluted with pentane, pentane–dichloromethane (9:1; 8:2; 7:3 and 1:1), dichloromethane–pentane (3:7); dichloromethane, dichloromethane–methanol (9:1; 7:3 and 1:1) and methanol, resulting in 29 fractions. Fractions 16 (6.6 mg) and 17 (11.7 mg) were identified by ^1^H, ^13^C, DEPT, HSQC, HMBC and NOESY, and by comparison with literature data [24]. Thus, fraction 16 was elucidated as a new compound, identified as *9β,13β-epoxy-7-abietene* (**1**) Amorphous white solid; GC retention time 33.78 min. ^1^H-NMR and ^13^C-NMR (Table 1). EI, *m/z* (rel. int.): 288 [M]^+^ (20), 161(100). Fraction 17 was identified as 6,7-didehydroroyleanone (**2**), already described by Kusumoto *et al.* [20]. 

### 3.5. Cytotoxicity *In Vitro*

The cytotoxicity assays were evaluated by the MTT colorimetric technique [19]. Analyses of cytotoxicity were performed at the Laboratory of Experimental Oncology of the Federal University of Ceará, Brazil. The used cell lines were MDA-MB-435, HCT-8 and SF-295 provided by the NCI (USA). The cells were grown in a medium consisting of 10% fetal calf serum in Roswell park memorial institute medium-1640 (RPMI-1640) supplemented with 1% antibiotic (100 U mL^−1^ of penicillin and 100 µg mL^−1^ of streptomycin). Cells were plated in a 96-well flat-bottomed plate with 0.1 × 10^6^ cells mL^−1^ per well for SF-295; MDA-MB-435 and 0.3 × 10^6^ cells per well for HCT-8. After 24 h incubation, the essential oil and isolated fractions were diluted in dimethyl sulfoxide (DMSO) using concentrations of 50 μg mL^−1^ for the essential oil and 25 μg mL^−1^ for the isolated fractions, and added to the wells in the plate. Next, the plates were incubated for 72 h at 37 °C in a humidified incubator with 5% of CO_2_. The supernatants were removed from the wells and cell viability evaluated using the MTT technique. After, 150 μL MTT solution was added [19] and the plates incubated for 3 h at 37 °C.

The plates were incubated for 1.5 h at 37 °C, and 150 μL of DMSO was added to the wells to dissolve the MTT crystals. The plates were placed on a shaker for 15 min and the absorbance was determined at 595 nm [18,21]. The percentage of cell growth was calculated by comparing the absorbance of test samples with the vehicle control (100%). The experiments were carried out in duplicate, repeated at least three times, and analyzed according to the average ± standard deviation of the percentage of inhibition of cell growth using the GraphPad Prism software.

### 3.6. Antioxidant Activity

The antioxidant activities were determined by DPPH radical scavenging, β-carotene-linoleic acid and 2,2'-azinobis-(3-ethylbenzothiazoline-6-sulfonic acid) (ABTS) assays. For the DPPH assay *T. riparia* essential oil or the 6,7-dehydroroyleanone was sequentially diluted and, for each dilution, 0.1 mL was mixed with a fresh DPPH methanolic solution (2.9 mL, 60 µM)—absorbance previously adjusted to 0.7 ± 0.05—to establish a curve [25,26]. After 30 min the decreasing value of absorbance at 515 nm was observed. In the β-carotene/linoleic acid system, the antioxidant activity was determined on the basis of the oxidation of β-carotene induced by the oxidative degradation of linoleic acid. This assay was described by Kumaran and Karunakaran [27]. β-carotene was dissolved in chloroform (0.2 mg mL^−1^) and an aliquot (2 mL) of this solution was transferred to a 100 mL flask. The chloroform was evaporated at room temperature, and then, linoleic acid (20 μL) and Tween 80 were added. To this solution, hydrogen peroxide (100 mL, distilled water treated with O_2_) was added, with vigorous stirring. An emulsion of β-carotene/linoleate was added to 0.2 mL of *T. riparia* essential oil and 6,7-dehydroroyleanone at different concentrations. The emulsion was placed in a water bath at 50 °C for 2 h; subsequently it was cooled down and the absorbance was read at 470 nm. For ABTS the free radical was prepared by mixing ABTS stock solution (7 mM in water) with 2.45 mM potassium persulfate. This mixture was kept during 16 h at room temperature in the dark. For ABTS radical assay, *T. riparia* essential oil or the isolated fraction was diluted and, for each dilution, 0.1 mL was mixed with 2.9 mL of ABTS methanolic solution—absorbance previously adjusted to 0.7 ± 0.05—to establish a curve. The reduction of absorbance was performed after 6 min at 734 nm [28]. The positive controls were standard solutions of quercetin (60 μM) [26] and BHT (900 μM).

The IC_50_ value—defined as the concentration of antioxidant compound that reduces 50% of the free radical concentration—was obtained by interpolation from linear regression analysis. Afterwards, results were analyzed by variance analyses and the differences among averages determined by Tukey’s test (*p* ≤ 0.01).

## 4. Conclusions

By fractioning the oil, it was possible to identify two compounds, an unpublished 9β,13β-epoxy-7-abietene with high cytotoxic potential, and an 6,7-dehydroroyleanone with high antioxidant potential. The essential oil and 9β,13β-epoxy-7-abietene showed high cytotoxic potential of the cell lines SF-295 (78.06% and 94.80%, respectively), HCT-8 (85.00% and 86.54%, respectively) and MDA-MB-435 (59.48% and 45.43%, respectively). 6,7-Dehydroroyleanone had no cytotoxic activity. The inhibitory concentrations (IC_50_ in µg mL^−1^) for essential oil and compound 6,7-dehydroroyleanone were: 15.63 and 0.01 for DPPH; 130.1 and 109.6 for β-carotene-linoleic acid and 1,524 and 1,024 for ABTS, respectively. The 9β,13β-epoxy-7-abietene had no antioxidant activity. The screening of natural products can provide greater structural diversity and offers significant opportunities for finding novel compounds. The compound 9β,13β-epoxy-7-abietene found in *T. riparia* essential oil is a new natural product, and its chemical structure is unprecedented in the literature. These results provided additional perspectives to the evaluation of new compounds with pharmacological activity. In future research we intend to expand the studies on biological activities of these compounds and other isolated fractions from the essential oil of *T. riparia* to develop new applications in pharmacology.
